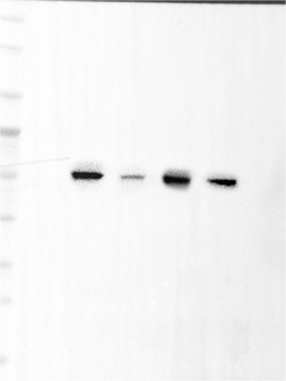# Paeoniflorin increases the anti-tumor efficacy of sorafenib in tumor-bearing mice with liver cancer via suppressing the NF-κb/PD-l1 axis

**DOI:** 10.1016/j.heliyon.2024.e24461

**Published:** 2024-01-13

**Authors:** Junfei Li, Chenghui Zhu, Zengyu Zhang, Xiaorong Zheng, Chunlei Wang, Hongyan Zhang

**Affiliations:** a Zhejiang Cancer Hospital, Hangzhou Institute of Medicine and Cancer (HIM), Chinese Academy of Sciences, Hangzhou, Zhejiang, 310022, China; b Wannan Medical College, Wuhu, Anhui, 241000, China; c The Second School of Clinical Medicine, Zhejiang Chinese Medical University No. 548, Binwen Road, Binjiang District, Hangzhou, Zhejiang, 310053, China

**Keywords:** Sorafenib, Hepatocellular carcinoma, NF-κB, PD-L1, Paeoniflorin

## Abstract

**Background:**

Sorafenib (Sor) represents a first-line therapy for hepatocellular carcinoma (HCC); however, its efficacy is constrained by secondary failure, which limits its clinical use. Recent studies have indicated that the suppression of Programmed cell death-Ligand 1 (PD-L1) may potentiate Sor's anti-liver cancer effects; furthermore, PD-L1 expression is known to be regulated by NF-κB. Previous research has demonstrated that paeoniflorin (PF) downregulates the NF-κB axis, nevertheless, current research has not yet determined whether PF can synergistically enhance the efficacy of Sor against HCC by modulating the NF-κB/PD-L1 pathway.

**Methods:**

The study employed a H22 hepatoma-bearing mouse model, which was treated with PF, Sor, and their combination over a period of 12 days. The impact of PF and Sor on tumor growth, proliferation, apoptosis, T-cell subsets, IL-2 and IFN-γ production, and NF-κB and PD-L1 expression was assessed. Moreover, Splenic lymphocyte from normal mice and tumor cells from model mice were co-cultured in vitro, and the tumor-specific cytotoxic T lymphocyte activity was analyzed. In the final phase of the study, Huh-7 cells were stimulated with PF in combination with an NF-κB activator or inhibitor, and the subsequent production of NF-κB and PD-L1 was investigated.

**Results:**

PF and Sor exhibit a synergistic anti-tumor effect, compared to the use of Sor alone, the combined use of PF and Sor significantly increased the number of CD4^+^ and CD8^+^ T cells in tumor tissue, markedly enhanced the cytotoxic activity of tumor-specific cytotoxic T lymphocytes, and reversed the depletion of interleukin-2 and the increase in PD-L1 expression following Sor intervention. This combination also further reduced the level of IFN-γ in peripheral blood and the expression of NF-κB and PD-L1 in tumor tissue. Additionally, in vitro experiments confirmed that PF reduces the expression of PD-L1 in Huh-7 liver cancer cells by inhibiting NF-κB.

**Conclusions:**

PF plays a synergistic role of Sor inhibiting HCC progression by regulating the NF-κB/PD-L1 pathway.

## Abbreviations

***HCC***: Hepatocellular carcinoma
***Sor***: Sorafenib
***TME***: IC
***NF-κB***: nuclear factor kappa-B
***PD-L1***: Programmed cell death-Ligand 1
***ICI***: Immune check point inhibitors
***PF***: Paeoniflorin
***CDI***: Combination Drug Index
***AOD***: Average optical density
***ELISA***: Enzyme linked immunosorbent assay
***IL-2***: Interleukin-2
***IFN-γ:*** Interferon-gamma
***CTLs***: Cytotoxic T lymphocytes
***OD***: Optical density
***CER***: Ceruletide

## 1 Introduction

Hepatocellular carcinoma (HCC) is a malignancy characterized by significant recurrence and mortality rates [1,2]. It has become the second most common cause of cancer-related mortality globally, often manifesting in individuals with chronic liver inflammation caused by viral infection, excessive alcohol consumption, or metabolic syndrome [3,4]. Significant advancements have been made in both the prevention and treatment of HCC. Regrettably, the majority of patients in the early stages of HCC are asymptomatic. Consequently, 80 % of HCC patients receive their diagnosis at advanced stages, and 70 % encounter a relapse within the initial five years following treatment [5]. Treatment for early-stage HCC typically involves radiofrequency ablation, liver transplantation, or resection. However, the treatment for advanced HCC typically necessitates Sorafenib (Sor) for systemic therapy, yet the desired outcomes have not yet been fully achieved [6].

Sor, a small-molecule tyrosine kinase inhibitor administered orally, is utilized as a primary treatment for advanced primary HCC. It significantly impacts the suppression of hepatoma cell proliferation, facilitates anti-tumor apoptosis, and inhibits metastatic activity, thereby offering hope to liver cancer patients [7,8]. However, prolonged use of Sor may result in a marked reduction in its efficacy, potentially impeding its therapeutic effectiveness in HCC [9,10]. Clinical trials indicate that patients using Sor monotherapy over prolonged periods do not exhibit significant prognostic improvements; their recurrence-free survival rate does not markedly increase, and their median survival duration diminishes [[11], [12], [13]]. Consequently, it is imperative to identify a safe and effective treatment that enhances the drug sensitivity of liver cancer to Sor for patients afflicted with this condition. The endeavor forms a principal focus of our current research efforts.

Currently, the reduction in efficacy of Sor is believed to involve a multi-step process, though the precise mechanism underlying desensitization is only partially elucidated, with novel treatments being actively investigated [14,15]. Recently, the role of the tumor microenvironment (TME) in various malignancies has attracted significant attention [16,17]. TME refers to the non-cancerous cells and components present within a tumor, playing a critical role in promoting tumor initiation, progression, metastasis, and response to treatment. There are complex interactions between cancer cells and the TME, crucial for the development of HCC, as the immune cells in the TME can exert antitumor effects [18]. Previous research has demonstrated that tumor-associated neutrophils recruit macrophages and Regulatory T cells (Tregs) to contribute to HCC cells' secondary failure to Sor [19]. In Sor-treated HCC patients, there were significant differences in the expression and composition of immune cells between sensitization and desensitization groups [20]. Programmed cell death-Ligand 1- (PD-L1) serves as a biomarker for immune checkpoint inhibitors (ICI) and plays a vital role in anti-cancer immune function [21,22]. When PD-L1, which is highly expressed on tumor cells, binds to the Programmed cell death ligand (PD-1) on T cells, it transmits a negative regulatory signal that suppresses the body's immune response. Inhibiting the PD-1/PD-L1 pathway can significantly boost the infiltration of CD4^+^ and CD8^+^ T cells in tumor cells, and effectively regulate the expression of a range of immune-related factors such as IL-2, IFN-γ, and TNF-α, thereby strengthening the body's anti-tumor immune function. A recent study demonstrated that knocking out the PD-L1 gene and suppressing PD-L1 production significantly improved Sor's therapeutic effect on liver cancer cells [23].

Paeoniflorin (PF), classified as a monoterpene glucoside, is the principal bioactive component of *Paeonia lactiflora Pall* [24]. PF is widely used in the treatment of numerous diseases due to its various pharmacological properties, such as anti-inflammatory, analgesic, immune regulation, as well as liver and nerve protection, cognitive impairment improvement, and anti-hyperglycemic effects [25,26]. PF demonstrates anti-tumor activity against non-small cell lung cancer, gastric cancer, liver cancer, and leukemia [27,28]. NF-κB is known to promote the transcription of the PD-L1 gene by binding to its promoter and to indirectly influence the post-transcriptional regulation of PD-L1. PD-L1 expression is positively modulated by NF-κB, establishing it as a pivotal factor in cancer progression [29]. PF has been demonstrated to suppress the transcription of nuclear factor kappa-B (NF-κB) and regulate immune function, thereby leading to the inhibition of tumor growth by decreasing NF-κB expression [30]. Given the crucial role of PD-L1 activation in Sor resistance and the impact of PD-L1 expression regulated by NF-κB, this study posits that PF can enhance Sor sensitization in HCC via the NF-κB/PD-L1 axis.

In this study, we investigated the anti-tumor effect and immunomodulatory activity of PF combined with Sor in an H22 hepatoma-bearing mouse model by evaluating tumor growth and analyzing the expression of Ki-67 (tumor proliferation), TUNEL (tumor apoptosis), as well as T-cell subsets of CD4^+^ and CD8^+^, IL-2 and IFN-γ production, and tumor-specific CTL response. Additionally, we investigated the effect of PF on the NF-κB/PD-L1 axis in liver cancer both in vivo and in vitro. This study aims to determine whether PF can enhance the anti-tumor immune function by suppressing NF-κB/PD-L1 axis， thereby improving Sor sensitivity in hepatomas.

## 2 Materials and methods

*Reagents:* Sor (purity >99.08 %), PF (purity >98.04 %), JSH-23 (purity >99.11 %), and Ceruletide (purity >99.84 %) were acquired from Medchemexpress Inc. (Shanghai, China). Antibodies against Ki-67 (ab15580) and NF-κB p65 (ab16502) (1:1000) were sourced from Abcam Inc. (Shanghai, China), while PD-L1/CD274 monoclonal antibody (66248-1-Ig) (1:2000) was acquired from Proteintech Group, Inc. (Wuhan, CN). CD4 monoclonal antibody (41-9766-82) and CD8 monoclonal antibody (42-0081-82) were procured from Thermo Fisher Scientific Inc. (Shanghai, China). Phosphate-buffered saline (PBS), sodium dodecyl sulfate-polyacrylamide gel electrophoresis (SDS-PAGE), and BCA protein assay kit were procured from Solarbio Biotechnology Co., Ltd. (Beijing, China), while fetal bovine serum (FBS) was obtained from Hyclone Bioscience Co., Ltd. (Beijing, China). A [3-(4,5-dimethylthiazol-2-yle)2,5-diphenyltetrazolium bromide] (MTT) kit was sourced from BD Biosciences (Franklin Lakes, NJ, USA), and mouse IL-2 and IFN-γ ELISA kits were acquired from Multi Science Biotechnology Co., Ltd. (Hangzhou, China). The TdT-mediated dUTP Nick End labeling (TUNEL) test kit was procured from Nanjing Jiancheng Biological Engineering Institute (Nanjing, China), and Dynabeads™ mouse Pan T were obtained from Thermo Fisher Scientific Inc. (Shanghai, China). Dulbecco's modified Eagle's medium (DMEM) was purchased from Gibco Biotechnology Co., Ltd. (Beijing, China), and PVDF membranes, dimethyl sulfoxide (DMSO), penicillin/streptomycin (PS), and 0.25 % Trypsin-0.53 mM EDTA were obtained from Wuhan Servicebio Technology Co., Ltd. (Wuhan, China).

*Cell line and culture.* The H22 mouse hepatoma cell line was obtained from Procell Life Science & Technology Co., Ltd. (Wuhan, China), while the Huh-7 human hepatoma cell line was acquired from iCell Bioscience Inc. (Shanghai, China). Following resuscitation, the cells were thawed in a water bath at 37 °C, following which DMEM high-glucose medium (containing 10 % FBS and 1 % PS) was introduced. The supernatant was discarded after centrifuging the mixture for 5 min at 1000 rpm. Following resuspension in complete medium, the cells were transferred to T25 culture flasks and incubated in a humidified environment with 5 % CO2 at 37 °C, with the medium being replaced every 24 h. Pancreatin was used to for digestion and passaging once approximately 80 % confluence was achieved.

*Animals and treatment. This study utilized* twenty-four C57BL/6 mice (5 weeks), each weighing between 18 and 22 g, which were obtained from SLAC ANIMAL Inc. (Shanghai, China). The animal experimentation was received approved by the Animal Experiment Ethics Committee of Zhejiang Cancer Hospital (Registration No.: SYKX 2017–0012, date of approval: 2017-10-10). Employing the Resource Equation Approach proposed by Arifin WN et al., the sample size for the animal experiment was calculated [31]. The mice were housed in a controlled environment with a 12-h light/dark cycle, with maintained temperatures at 22 ± 2 °C, and a relative humidity of 45 ± 10 %. For the establishment of the H22 hepatoma-bearing mice model, H22 cells (1 × 10^6^, with a viability exceeding 95 %) were subcutaneously injected into the necks or backs of the mice. After the tumor reached approximately 50 mm^3^, the model mice were randomly allocated to one of four groups, each consisting of six mice: a model group (0.2 mL normal saline, administered orally (i.g.) once daily), a Sor group (30 mg/kg Sor, administered orally once daily), a PF group (25 mg/kg PF [32], administered orally once daily), and a PF-Sor group (30 mg/kg Sor and 25 mg/kg PF, administered orally once daily).

Tumor dimensions, including the longest and the shortest diameters perpendicular to the longest diameter, were measured every two days using vernier calipers [33]. During measurement, care was taken to ensure that the jaws of the calipers gently touched the surface of the tumor, avoiding any pressure on the animal or the tumor. Drug administration and measurement tasks were independently conducted by two researchers. Tumor size measurements were performed bi-daily using calipers, and the tumor volume was calculated using the formula: volume (mm^3^) = length × width^2^/2. Throughout the entire. experimental period, the mice were provided ad libitum access to standard rodent food and water, with the intervention treatments extending over a duration of 12 days. At the conclusion of the 12-day treatment period, all surviving mice underwent euthanasia via cervical dislocation, and tumor tissues being promptly collected and weighed. The Combination Drug Index (CDI) calculation was performed to assess the synergistic impact of the combined drugs, utilizing the formula: CDI = AB/(A × B), where A or B represents the tumor weight in the single-drug group divided by the tumor weight in the model group, and AB signifies the tumor weight in the combination group divided by the tumor weight in the model group). A CDI value less than 1 indicates a synergistic effect, while a value less than 0.7 indicates a significant synergistic effect. Conversely, a CDI value equal to 1 denotes an additive effect, and a CDI value exceeding 1 indicates antagonism.

*Immunohistochemistry*. From each group, three samples were randomly selected, and an immunohistochemistry analysis was conducted to investigate the production of Ki-67, NF-κB, and PD-L1 in tumor tissue. Initially, the samples underwent deparaffinization and rehydration. Subsequently, either trypsin treatment for 10 min or heat treatment for 25 min was employed prior to the addition of the primary antibody. The slices were then placed flat in a wet box and incubated at 4 °C overnight. Following three washes, the secondary antibody was added, and the tissue was incubated at room temperature for 30 min. Thereafter, the 3,3N-Diaminobenzidine Tertrahydrochloride (DAB) color developing solution was added, followed by hematoxylin for counterstaining. Ultimately, the samples were examined under a microscope for images acquisition. Image-pro Plus 6.0 software was utilized to calculate the average optical density (AOD).

*Immunofluorescence.* For examining the tissue sections, the samples were dewaxed and rehydrated, then placed in a blocking solution. Following a 1-h block at room temperature, the primary antibody was subsequently added and incubated overnight at 4 °C. Upon removal of the blocking solution, the secondary antibody was then added and incubated at room temperature for 30 min. Subsequently, the 4′,6-diamidino-2-phenylindole (DAPI) stain was added and incubated at room temperature for 10 min, following by sealing with an anti-fluorescence quenching agent. Following microscopic examination, images were captured, and the AOD was calculated using Image-pro Plus 6.0 software.

*TUNEL assay.* The tissue sample underwent dewaxing and was hydrated with ethanol. Subsequently, an appropriate amount of TUNEL balance solution was added and the sample was incubated for 10–30 min. Thereafter, the TUNEL reaction mixture was applied to cover the tissue section. Next, the DAPI stain solution was carefully added dropwise, and the sections were subsequently incubated in the dark at room temperature for 10 min. Ultimately, the sections were sealed with an anti-fluorescence quenching agent. Subsequently, the sections were examined under a fluorescence microscope and images were captured. The ratio of apoptotic cells to total cells was quantified using Image-pro Plus 6.0 software.

*Enzyme Linked Immunosorbent Assay (ELISA)*. Peripheral blood obtained from mice underwent centrifugation for 10 min at 3000 rpm to extract serum. The ELISA technique was utilized to measure the concentrations of interleukin-2 (IL-2) and interferon-gamma (IFN-γ) in the serum, adhering to the manufacturer's instructions (Multi Science Biotechnology Co., China). Subsequently, the absorbance value was determined using a microplate reader.

*Assessment of Tumor-Specific Cytotoxic T Lymphocyte Response.* For analyzing the tumor-specific cytotoxic activity of cytotoxic T lymphocytes (CTLs), the MTT assay was utilized [34]. Splenic lymphocytes derived from normal mice were cultured in 96-well plates for up to 24 h until adherence, serving as effector cells. Tumor cells harvested from each experimental group of mice served as target cells. Effector and target cells (3 × 10^4^/well, 100 μL/well) were seeded into 96-well plates in ratios of 10:1, 20:1, and 40:1 for the experimental groups, respectively. Wells containing effector cells and culture medium (100 μL/well) constituted the effector groups, while those containing target cells and culture medium (100 μL/well) formed the target groups. Following a 20 h period, 20 μL of MTT solution (5 mg/mL) was added to the cells, which were then incubated for an additional 4 h at 37 °C. Subsequently, the cells were resolved with 200 μL of DMSO and the optical density (OD) at 490 nm was measured using a microplate reader (Varioskan Flash, Thermo) to determine the OD value. Upon subtracting the background, the tumor-specific cytotoxic activity was calculated using the formula: [100 % × (OD _target group_ − (OD _experimental group_ − OD _effector group_)/OD _target group_)].

*Cell Viability Assay.* Huh-7 cells, in the logarithmic growth phase, were seeded in 96-well plates and incubated for 24 h to allow adherence. Subsequently, the cells were treated with various concentrations of PF (0, 5, 10, 20, 40, and 80 μM) [35] and then incubated for an additional 24 h. After the addition of the MTT solution (20 μL), the cells were further incubated for 4 more hours at 37 °C. Absorbance values were measured at 450 nm. Cell viability was calculated using the formula below after subtracting the background: cell viability (%) = [ A_450__(drug)_/A_450 (control)_ × 100 %]. Data were presented as a dose-response curve. The Cytotoxic concentration 10(CC10) value, indicative of the concentration needed to reduce cell viability to 10 %, was determined through nonlinear regression analysis. The goodness of fit for the curve was assessed and the CC10 value, along with its 95 % confidence interval, was reported.

*Western Blot Assay.* Huh-7 cells were treated with various concentrations of PF (5, 10, 20 μM) and were subsequently cultured for 24 h. Furthermore, Huh-7 cells were pre-treated with either an NF-κB inhibitor, JSH-23 (30 μM), or an NF-κB activator, Ceruletide (CER, 1 μM), for 2 h, followed by co-treatment with PF (20 μM) for 24 h [36]. Protein samples isolated from cells were normalized using a BCA kit and subsequently loaded onto SDS-PAGE (8–12 %) gels, which were thereafter transferred to a PVDF membrane and blocked. The primary antibody was then incubated with the PVDF membrane overnight at 4 °C. The membranes were then washed with Tween-Tris-buffered saline solution and then treated with a secondary antibody at room temperature for 2 h. Blotting visualization was performed using the chemiluminescence method, adhering to the manufacturer's instructions. β-actin served as an internal reference, and the gray value of the bands was quantified using Image J software.

*Statistical Analysis.* Data were presented as the mean ± standard error of the mean (SEM). A one-way analysis of variance (ANOVA) was used to compare multiple groups，followed by a Tukey multiple comparisons test. A significance level of P < 0.05 was considered to statistical significance. Statistical analyses were performed using GraphPad Prism 8.0.

## 3 Results

*In H22 tumor-bearing model mice，the combination of PF and Sor was found to exhibit an anti-hepatoma effect.* Following the establishment of the model, treatments with either PF, Sor, or their combination commenced on the 6th day, with no significant difference in tumor size among groups at the onset of intervention. After 12 days of treatment with different drugs, compared to the model group, the PF-treated hepatoma-bearing mice exhibited a significant reduction in tumor volume and weight (P < 0.05), while the Sor-treated and combination therapy groups showed a highly significant decrease in both tumor volume and weight (P < 0.01). Furthermore, compared to the sole use of Sor, the combination of PF and Sor exhibited a more significant anti-tumor effect (P < 0.05, CDI = 0.89). These findings indicate that PF and Sor, when used in combination, have a synergistic effect in combating liver cancer. (Fig. 1 A & B).

**Fig. 1:**
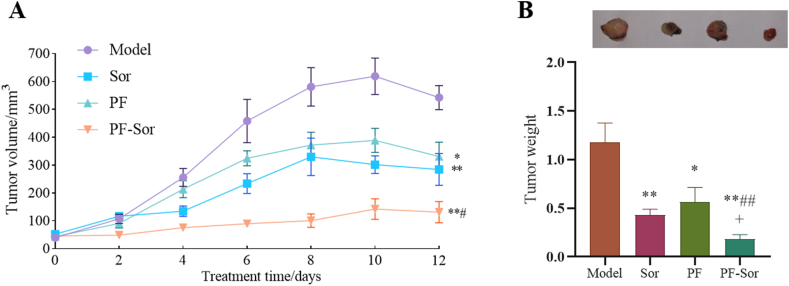
The anti-hepatoma effect of paeoniflorin (PF) combined with Sorafenib (Sor) in an H22 hepatoma-bearing mice model. (A) shows a significant decrease in tumor volume when PF and Sor were combined (n = 6，1-way ANOVA, with a Tukey post test.). (B) demonstrates a substantial reduction in tumor weight when PF was coupled with Sor (n = 6，1-way ANOVA, with a Tukey post test.). Data are presented as the mean ± SEM. *p < 0.05 compared to the model group; **p < 0.01 compared to the model group; #p < 0.05 compared to the Sor group; ##p < 0.01 compared to the Sor group. In addition, ^+^CDI<1 indicates a synergistic effect when the two drugs were combined.

*Effect of PF Combined with Sor on Proliferation and Apoptosis in H22 Hepatoma Tumor-Bearing Mice In Vivo*. To assess the impact of PF combined with Sor on tumor cell proliferation and apoptosis, the level of Ki-67 in tumor tissues was measured, and apoptosis was evaluated using TUNEL assays. Compared to the model group, a marked reduction in Ki-67 expression was observed in the tumor tissues of the PF group, Sor group, and the combination therapy group, with a progressive enhancement in the inhibition of tumor proliferation (P < 0.01). Notably, the anti-proliferative effect of the combination therapy was significantly stronger than that of the Sor group alone (P < 0.01) (Fig. 2A). Furthermore, the quantity of TUNEL-fluorescent cells in the model group was significantly lower compared to both the Sor group and the PF plus Sor combination group (P < 0.05). The combination of PF and Sor yielded a high number of positive cells than the Sor group alone (P < 0.05) (Fig. 2B). These findings demonstrate that PF augments the inhibitory effect of Sor on proliferation and promotes apoptosis in H22 hepatoma-bearing mice.

**Fig. 2:**
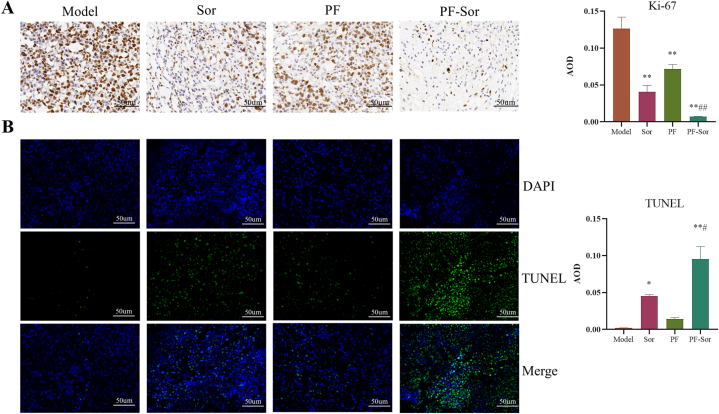
Impact of paeoniflorin (PF) Combined with Sorafenib (Sor) on Proliferation and Apoptosis in H22 Hepatoma-Bearing Mice Tumor Tissue. (A) The expression regions of Ki-67 were stained brown. Ki-67 production was significantly decreased in the Sor, PF, and combination treatment groups (n = 3，1-way ANOVA, with a Tukey post test.). (B) The expression regions of DAPI were stained blue, and the expression regions of TUNEL were stained green. The PF combined with Sor treatment resulted in increased apoptotic cell numbers in tumor samples compared to the modell group (n = 3，1-way ANOVA, with a Tukey post test.). Data are displayed as the mean ± SEM. **p < 0.01 relative to the model group; #p < 0.05, ##p < 0.01 relative to the Sor group.

*Impact of PF Combined with Sor on Anti-Tumor Immunity in Model Mice.* To determine if the anticancer effect of PF combined with Sor resulted from enhanced immunological responses, the infiltration of T-cell subsets in tumor tissue, levels of IFN-γ and IL-2 in peripheral blood, and CTL activity of normal mice's Splenic lymphocyte against model mice's tumor cells were examined. Compared to the model group, Sor significantly promoted CD4^+^ T-cell infiltration in the tumor tissues of tumor-bearing mice (P < 0.05), with the PF group and the combination therapy group exhibiting even stronger effects than the Sor group (P < 0.01). Conversely, Sor significantly reduced CD8^+^ T-cell infiltration in tumor tissues compared to the model group (P < 0.05). However, treatment with PF, as well as the combination therapy, significantly enhanced CD8^+^ T-cell infiltration in tumor tissues of tumor-bearing mice (P < 0.01) These findings indicate that PF enhanced T-cell infiltration in tumor tissues following Sor treatment (Fig. 3 A & B).

**Fig. 3:**
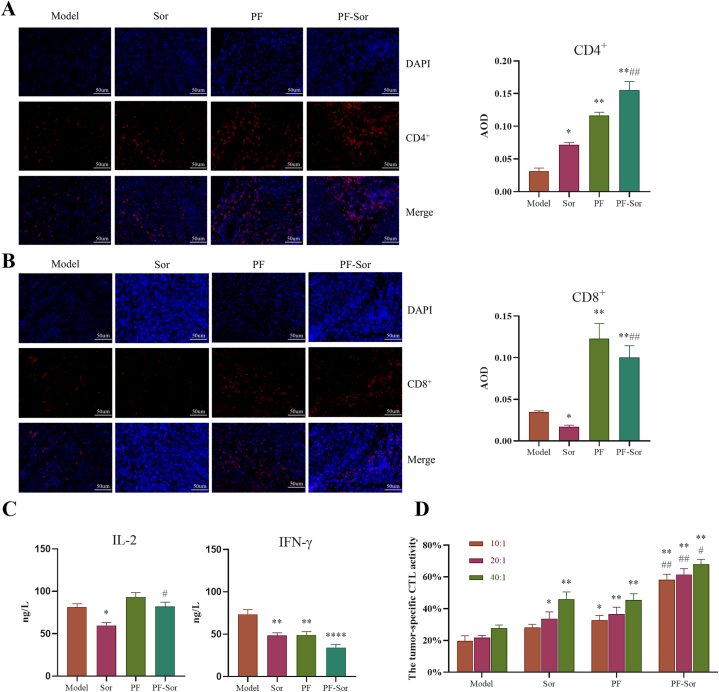
paeoniflorin (PF) combined with Sorafenib (Sor) on anti-tumor immunity impact of H22 tumor-bearing mice. (A) (B) The expression regions of DAPI were stained blue, and the expression regions of CD4^+^ or CD8^+^ cells were stained red. The infiltration of CD4^+^ and CD8^+^ cells in tumor tissue and the AOD were quantified (n = 3, 1-way ANOVA, with a Tukey post test). (C) Effect of PF in combination with Sor on IFN-γ and IL-2 production in peripheral blood of mice (n = 6, 1-way ANOVA, with a Tukey post test). (D) The ability of normal mouse Splenic lymphocyte to kill drug-treated mice tumor cells (n = 6, 1-way ANOVA, with a Tukey post test). Data are displayed as the mean ± SEM. *p < 0.05 relative to the model group; **p < 0.01 relative to the model group; ****p < 0.0001 relative to the model group; #P < 0.05 relative to the Sor group; ##p < 0.01 relative to the Sor group;

Levels of IFN-γ and IL-2 in the peripheral blood of the tumor-bearing mice in each group were measured. Compared to the model group, Sor treatment significantly reduced IL-2 levels in the peripheral blood of tumor-bearing mice (P < 0.05). While PF or the combined treatment increased IL-2 levels, the difference was not significant. However, the IL-2 level in the combination group was significantly higher than in the Sor group alone (P < 0.05). On the other hand, compared to the model group, intervention with Sor and PF significantly reduced IFN-γ levels in the peripheral blood of tumor-bearing mice (P < 0.01), with no significant difference between the two. However, the combined treatment group exhibited a stronger inhibition compared to either Sor or PF alone（P < 0.0001）(Fig. 3C).

Lymphocytes isolated from the spleens of normal mice were co-cultured with tumor cells isolated from the tumor tissues of each experimental group of tumor-bearing mice. At a co-culture ratio of 10:1, compared to the model group, the cytotoxicity of splenic lymphocytes against tumor cells from the Sor group showed no significant difference, but significantly increased against the PF group's tumor cells (P < 0.05) and was highly significant against the PF-Sor group's tumor cells (P < 0.01). At a co-culture ratio of 20:1, splenic lymphocytes showed a significant increase in cytotoxicity against Sor group tumor cells (P < 0.05) compared to the model group, and highly significant increases against tumor cells of both the PF and PF-Sor groups (P < 0.01). At a co-culture ratio of 40:1, the cytotoxicity of splenic lymphocytes against tumor cells from all groups was highly significantly increased compared to the model group (P < 0.01). Additionally, across all ratios, the cytotoxicity of splenic lymphocytes against tumor cells from the PF-Sor group was significantly higher than against those from the Sor group (P < 0.05). This suggests that the combined intervention of PF and Sor enhances the specific cytotoxic activity of lymphocytes against tumor cells (Fig. 3D).

*Impact of PF Combined with Sor on the Expression of NF-κB and PD-L1 in Tumor Tissues.* To investigate the impact of the NF-κB/PD-L1 axis, the protein expression levels of PD-L1 and NF-κB in tumor tissues were measured. Results indicated a significant reduction in NF-κB levels in the tumor tissues of the Sor, PF, and PF-Sor groups compared to the model group, with the PF-Sor group showing notably lower NF-κB production than the Sor group. Furthermore, treatment with Sor alone significantly increased PD-L1 levels compared to the model group, whereas treatment with PF alone had a contrasting effect, effectively reversing the increase in PD-L1 expression caused by Sor intervention. (Fig. 4).

**Fig. 4:**
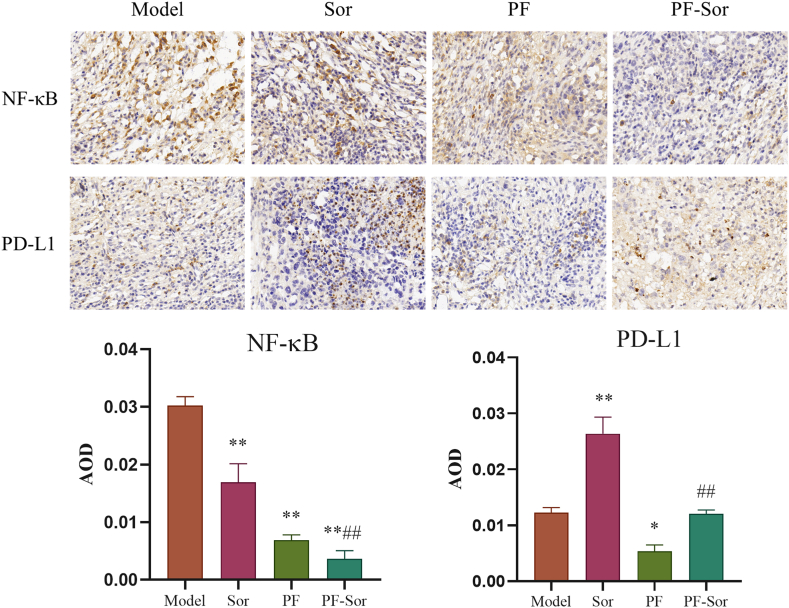
paeoniflorin (PF) combined with Sorafenib (Sor) on the expression of NF-κB and PD-L1 in tumor tissues (n = 3, 1-way ANOVA, with a Tukey post test). The expression regions of NF-κB or PD-L1were stained brown. Data are displayed as the mean ± SEM. *p < 0.05 relative to the model group; **p < 0.01 relative to the model group; ##p < 0.01 relative to the Sor group;

*Effect of PF on the Viability of Huh-7 Cells.* To investigate the effect of PF on the viability of Huh-7 cells, the cells were treated with varying doses (0, 5, 10, 20, 40, and 80 μM) of PF. Subsequently, a dose-response curve was constructed, and the CC10 value (maximum non-toxic concentration) was determined to be 22.96 μM through nonlinear regression analysis (Fig. 5).

**Fig. 5:**
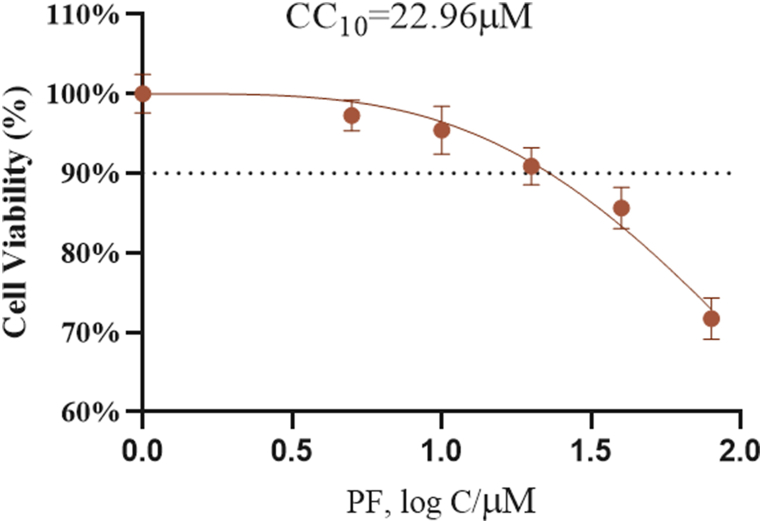
Dose response curves of Huh-7 cell viability under treatment with different concentrations of paeoniflorin (0, 5, 10, 20, 40, and 80 μM) for 24 h. The CC_10_ values was calculated using non-linear regression analysis. Data are displayed as the mean ± SEM.

*PF inhibits PD-L1 expression through the down-regulation of NF-κB in Huh-7 cells.* To determine whether PF reduces PD-L1 expression in Huh-7 cells via NF-κB, protein levels of NF-κB and PD-L1 were assessed using Western blot analysis. In Comparison to the control group, Huh-7 cells exposed to three non-toxic concentrations of PF (5, 10, and 20 μM) exhibited reduced expression of NF-κB (P65) and PD-L1 (Fig. 6A). To further elucidate the role of PF in down-regulating NF-κB to inhibit PD-L1 expression, Huh-7 cells were co-treated with NF-κB inhibitor (JSH-23) and activator (CER) in combination with PF. The results indicated that JSH-23 enhances the inhibitory effect of PF (20 μM) on PD-L1 expression in Huh-7 cells, while CER partially reversed the suppression of PD-L1 protein expression induced by PF(20 μM) in Huh-7 cells. These findings suggest that PF reduces PD-L1 levels by down-regulating NF-κB (Fig. 6 B & C).

**Fig. 6:**
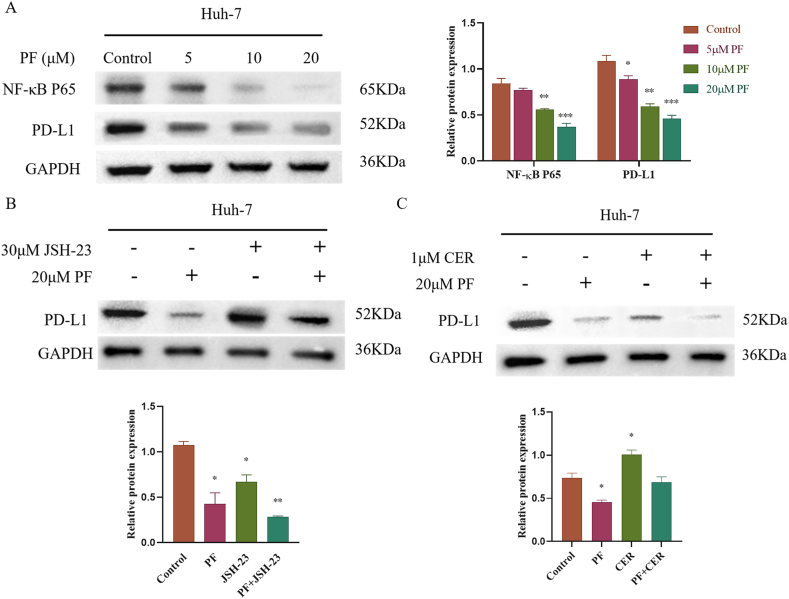
paeoniflorin (PF) inhibiting PD-L1 expression by down-regulation NF-κB in Huh-7 cells. (A) Effects of PF on the NF-κB and PD-L1 levels in Huh-7 cellls. Western-blot was performed to measure the relative protein expression of NF-κB and PD-L1 (n = 3, 1-way ANOVA, with a Tukey post test). (C) The expression of PD-L1 was analyzed by Western-blot after pretreatment PF combination with JSH-23 orCER in Huh-7 Cells (n = 3, 1-way ANOVA, with a Tukey post test). Data are displayed as the mean ± SEM. *p < 0.05 relative to the control group; **p < 0.01 relative to the control group; ***p < 0.001 relative to the control group.

## 4 Discussion

The activation of the NF-κB pathway is known to reduce the efficacy of certain anticancer therapies, including chemotherapy and radiation, and is implicated in Sor desensitization in HCC [37,38]. Previous studies have shown that PF can down-regulate the expression of NF-κB, and NF-κB is known to promote PD-L1 expression [29,30]. Therefore, we investigated the efficacy of combined therapy using PF and Sor to improve the treatment of HCC through the NF-κB/PD-L1 axis.

We investigated the potential anti-tumor effect of the combination of PF and Sor in H22 hepatoma tumor-bearing mice. The results indicated that the combination of PF and Sor significantly reduced tumor growth, as evidenced by decreased tumor volume and weight, compared to Sor alone. Additionally, a synergistic effect was observed when PF was combined with Sor. Notably, PF enhanced the ability of Sor to induce apoptosis and inhibit tumor cell proliferation in tumor tissue. These findings underscore the potential of PF in enhancing the therapeutic efficacy of Sor for HCC treatment. Furthermore, efforts were made to elucidate the underlying mechanism behind the synergistic effect of PF and Sor in HCC treatment.

Recent studies indicate that modifications in anti-tumor immune responses can significantly influence the effectivenese of Sor in treatment of hepatoma [39,40]. In this study, the effect of PF in combination with Sor on anti-tumor immune function was investigated in a hepatoma-bearing mouse model. T lymphocytes, comprising CD4^+^ and CD8^+^ T cells, play a pivotal role in generating and regulating tumor antigen-specific immune responses. CD4^+^ T cells, with various subtypes including CD4^+^ helper cells (Th), secrete a range of cytokines such as IL-2, IFN-γ, and TNF-α to promote CD8^+^ T cell proliferation and infiltration, enhancing their cytotoxicity against tumors. However, CD4^+^ regulatory T cells can suppress immune function, leading to IL-2 depletion, reducing CD8^+^ T cell infiltration and activity in tumor tissues, thus facilitating tumor cell immune escape [41,42]. Additionally, while the increased expression of IL-2 and IFN-γ facilitate the maturation and differentiation of immune cells, IFN-γ may also lead to PD-L1 expression in tumor cells, thereby leading to immune escape [43,44]. Our study demonstrated that after treatment with Sor, there was an observed increase in CD4^+^ T cell infiltration and a decrease in CD8^+^ T cell infiltration in the tumor tissues of tumor-bearing mice, accompanied by reduced expression of IL-2 and IFN-γ in peripheral blood, similar to the findings of previous findings [45]. The increased expression of Treg cells has been closely associated with Sor resistance [46]. The above results suggest that Sor intervention may lead to an increased infiltration of Treg cells instead of Th cells in tumor tissues, therebyinhibiting the infiltration and anti-tumor activity of CD8^+^ T cells. Nevertheless, compared with the sole use of Sor, the co-administration of PF and Sor in tumor-bearing mice significantly enhanced the infiltration of CD4^+^ T cells in tumor tissues, while reversing the inhibition of CD8^+^ T cell infiltration and reversing the IL-2 depletion induced by Sor. Simultaneously, compared to the Sor group, normal spleen lymphocytes demonstrated a significantly enhanced cytotoxicity against tumor cells following the combined drug intervention. These findings suggest that PF may enhance the anti-tumor efficacy of Sor by promoting the infiltration and anti-tumor immune activity of CD8^+^ T cells in tumor tissues through enhancing Th cells rather than Treg cells, consistent with previous studies on PF [47]. Moreover, PF significantly reduces IFN-γ expression in the peripheral blood of tumor-bearing mice following Sor intervention, potentially leading to the reduced PD-L1 expression in tumor tissues.

Studies have demonstrated that increased PD-L1 expression in the tumor microenvironment leads to immunosuppression, which facilitates both tumor immune escape and resistance to anti-tumor medication. For instance, the upregulation of PD-L1 has been associated with increase resistance to cisplatin in patients with small cell lung cancer [48], and resistance to BRAF inhibitors in patients with metastatic melanoma is similarly linked to PD-L1 [49]. Recent investigations suggest that increased PD-L1 expression following Sor intervention leads to liver tumor cell desensitization to Sor [50,51]. This aligns with our findings, where our study revealed that PF significantly reduces PD-L1 and NF-κB expression in the tumor tissues of Sor-treated tumor-bearing mice.

The previous research found that NF-κB interacts with the proximal region of PD-L1 promoter to facilitate PD-L1 expression in human cervical cancer cells [52]. In light of this evidence, we hypothesized that PF potentially enhances Sor's anti-hepatoma effect by inhibiting NF-κB expression and reducing the level of PD-L1 in tumor cells, thereby regulating anti-tumor immune function. Our work indicates that PF promotes the expression of PD-L1 and NF-κB in tumor tissues of hepatocellular carcinoma-bearing mice following Sor intervention. We also demonstrated in vitro that PF is capable of inhibiting the expression of NF-κB and PD-L1 in huh-7 cells in a dose-dependent manner. Moreover, the use of NF-κB inhibitors or agonists has been shown to enhance or weaken the effect of PF on PD-L1 expression. Both in vitro and in vivo experiments have demonstrated that PF decreases the amount of PD-L1 expression in tumor cells, mirroring the results of other studies [45]. PF may enhance the sensitivity of HCC to Sor therapy by reducing the expression of NF-κB, thereby inhibiting the levels of PD-L1 [23].

Lastly, there remain some unresolved issues in our research. We did not discuss whether the effect of PF in enhancing the anti-hepatocarcinoma action of Sor is dose-dependent. In addition, the regulatory effect of PF on Th or Treg cells also needs further elucidation. Furthermore, the mechanism through which PF regulates the NF-κB/PD-L1 axis remains to be elucidates. In our future research, we intend to further investigate and address these issues.

## 5 Conclusion

Our study demonstrated that PF regulates NF-κB and reduces the PD-L1 level in tumors, thereby enhancing anti-cancer immune function and significantly improving the sensitivity of HCC to Sor. This suggests that PF is a potential therapeutic agent to enhance the anti-hepatoma effect of Sor. Our research contributes new theoretical and experimental evidence supporting the clinical use of PF in combination with Sor to treat liver cancer. Additionally, it contributes to the understanding of integrating traditional Chinese medicine with Sor in treating liver cancer and potentially aids in improving treatment outcomes for liver cancer patients.

## Funding

This work was supported by the “10.13039/501100017594Medical Science and Technology Project of Zhejiang Province, China” under Grant No. 2020KY074 and the “10.13039/501100012175Zhejiang Traditional Chinese Medicine Administration, China” under Grant No. 2022ZA025 and the “10.13039/501100020777Zhejiang Pharmaceutical Association, China” under Grant No. 2021ZYY17.

## Consent for publication

Not applicable.

## Availability of data and materials

The data used to support the findings of this study are available from the corresponding author upon request.

## Ethical approval

Animal treatment and maintenance were performed in accordance with the Principles of Laboratory Animal Care (NIH Publication no.85–23, revised 1985). All procedures for animal care and experiments were reviewed and approved by the Zhejiang Cancer Hospital Animal Ethical Committee (No. zjzlsd 2021-07-198).

## Patient consent for publication

Not applicable.

## CRediT authorship contribution statement

**Junfei Li:** Writing – review & editing, Writing – original draft, Methodology, Investigation, Funding acquisition, Conceptualization. **Chenghui Zhu:** Writing – review & editing, Writing – original draft, Methodology. **Zengyu Zhang:** Writing – original draft, Methodology, Data curation. **Xiaorong Zheng:** Investigation. **Chunlei Wang:** Data curation. **Hongyan Zhang:** Writing – review & editing, Supervision, Conceptualization.

## Declaration of competing interest

The authors declare that they have no known competing financial interests or personal relationships that could have appeared to influence the work reported in this paper.

## Figures and Tables

**figs1:**
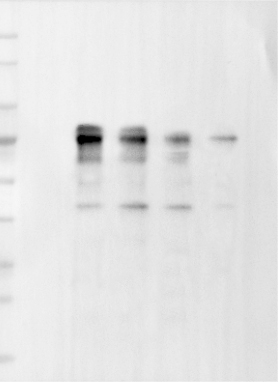


**figs2:**
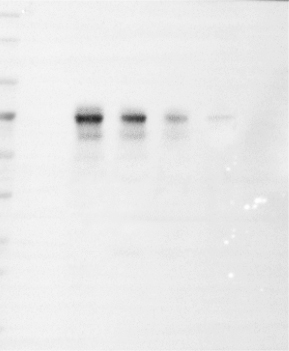


**figs3:**
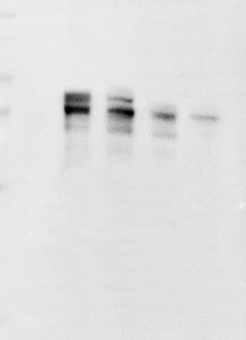


**figs4:**
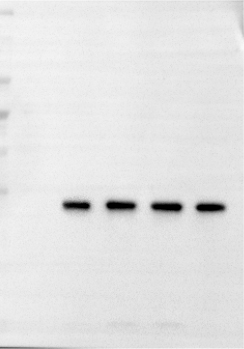


**figs5:**
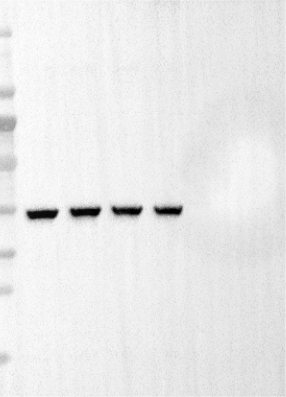


**figs6:**
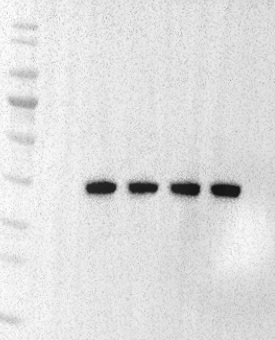


**figs7:**
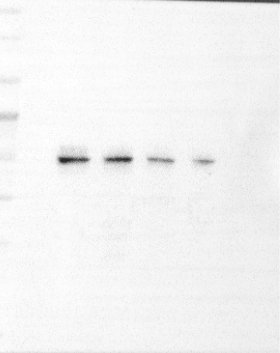


**figs8:**
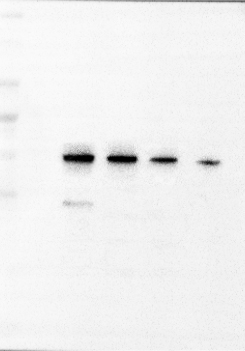


**figs9:**
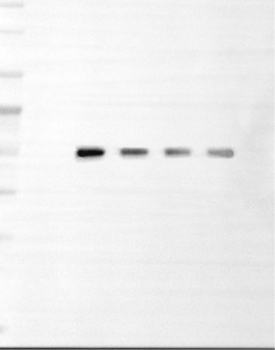


**figs10:**
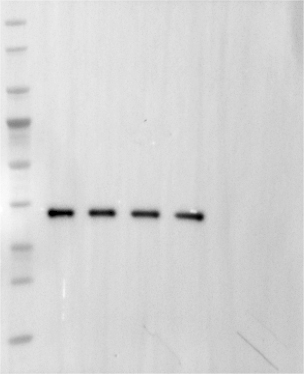


**figs11:**
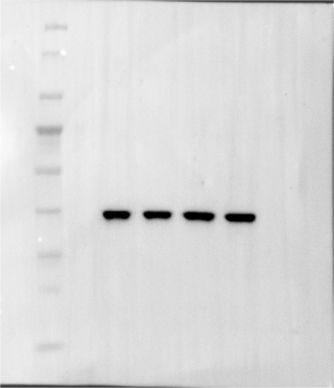


**figs12:**
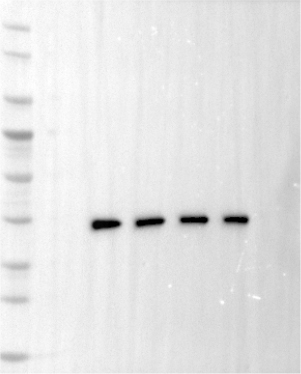


**figs13:**
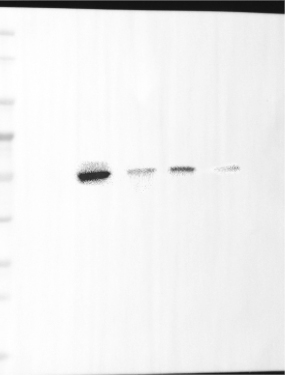


**figs14:**
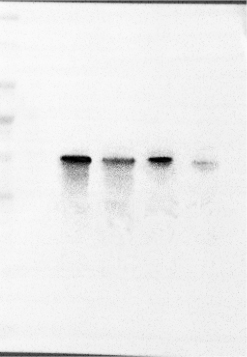


**figs15:**
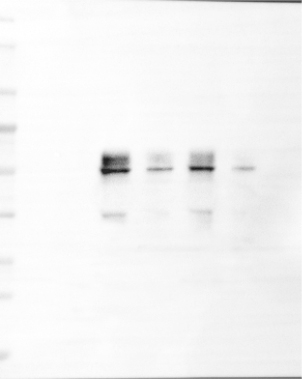


**figs16:**
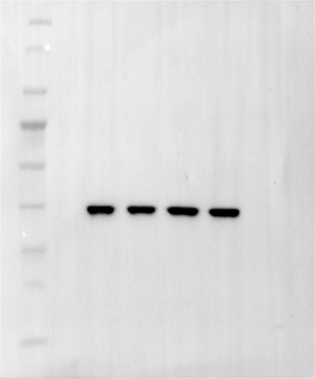


**figs17:**
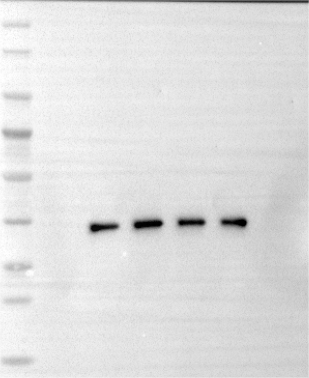


**figs18:**
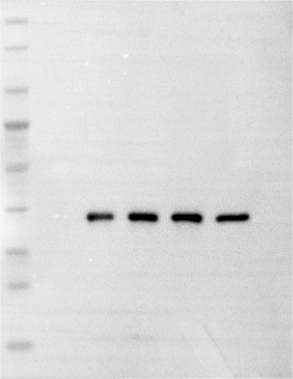


**figs19:**
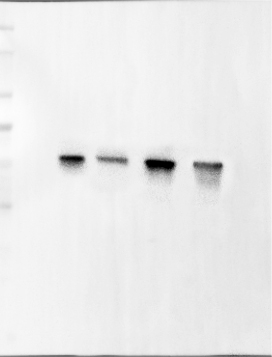


**figs20:**
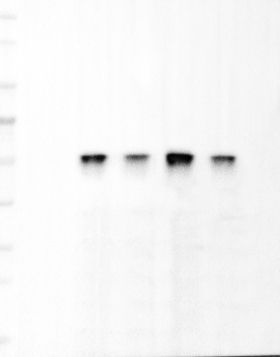


**figs21:**